# Association of hepatic steatosis with MRI-defined renal steatosis in type 2 diabetes: a cross-sectional imaging and clinical modeling study

**DOI:** 10.3389/fnut.2026.1881517

**Published:** 2026-07-31

**Authors:** Yuzhu Zhu, Futian Liu, Yina Zhang, Xiaorong Guo, Shaoling Yang, Jiangping Zeng, Zaifei Yin, Dan Guo, Wenxin Ju, Rong Sun, Hong Sun

**Affiliations:** 1 Department of Endocrinology and Metabolism, The Fourth Affiliated Hospital of Soochow University, Suzhou Dushu Lake Hospital, Medical Center of Soochow University, Suzhou, China; 2 Department of Endocrinology and Metabolism, Kunshan Hospital of Chinese Medicine, Suzhou, Jiangsu, China; 3 Department of Radiology, The Fourth Affiliated Hospital of Soochow University, Medical Center of Soochow University, Suzhou, Jiangsu, China

**Keywords:** clinical classification model, Dixon MRI, fatty kidney disease, metabolic dysfunction-associated fatty liver disease, type 2 diabetes

## Abstract

**Background:**

Fatty Kidney Disease (FKD) is increasingly recognized as a distinct clinical entity, often coexisting with metabolic dysfunction-associated fatty liver disease (MAFLD) in type 2 diabetes mellitus (T2DM). However, the strength of their association and the metabolic factors underlying this association remain incompletely characterized. We examined the cross-sectional association between MRI-derived hepatic and renal fat and developed a clinical model for classifying an MRI-defined renal steatosis phenotype.

**Methods:**

This single-center cross-sectional study included 257 adults with T2DM. Hepatic fat fraction (HFF) and renal fat fraction (RFF) were quantified using 3.0-T Dixon MRI. RFF ≥ 4% was used as an exploratory definition of renal steatosis, with sensitivity analyses at thresholds of ≥3% and ≥5%. Associations were assessed using correlation, multivariable regression, and restricted cubic splines. A classification model was developed using LASSO-assisted variable selection and logistic regression, with internal validation by 1,000 bootstrap resamples. XGBoost, SHAP, and exploratory cross-sectional indirect-effect analyses were also performed.

**Results:**

HFF showed the strongest correlation with RFF (r = 0.534, *p* < 0.001). MRI-defined fatty liver, fasting C-peptide (FCP), and high-sensitivity C-reactive protein (hsCRP) were retained in the final model. The apparent AUC was 0.858, and the optimism-corrected AUC was 0.833. At RFF thresholds of ≥3% and ≥5%, the AUCs were 0.827 and 0.784, respectively. Systolic blood pressure, uric acid, HDL-C, and hsCRP showed significant indirect effects, accounting for 4.62–15.16% of the observed HFF–RFF association.

**Conclusion:**

Hepatic steatosis was strongly associated with MRI-defined renal steatosis in individuals with T2DM. The three-variable model demonstrated good internally validated discrimination, with a bootstrap-corrected AUC of 0.833, although external validation remains necessary. Exploratory indirect-effect analyses suggested that inflammatory and metabolic factors may partially account for the observed association. These findings may inform the future development of risk-classification strategies and provide a basis for future longitudinal and mechanistic studies.

## Graphical abstract

**Figure:**
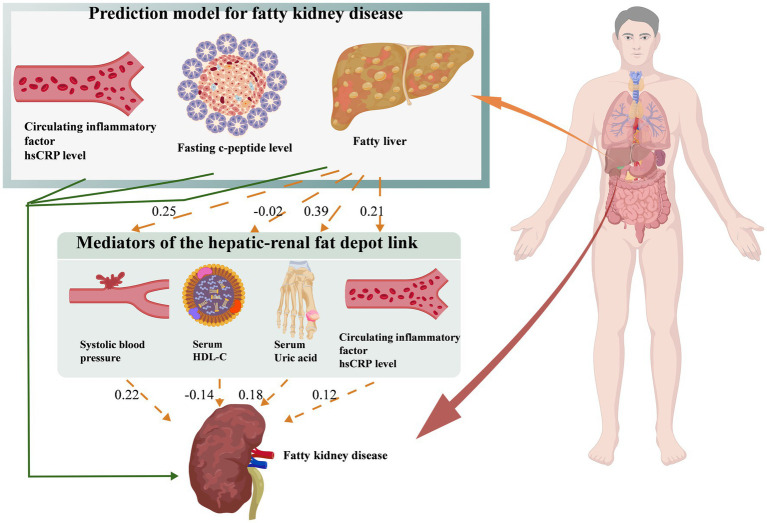
Multipath mechanisms of metabolic factors in fatty kidney disease. The model illustrates that fatty liver, fasting C-peptide (FCP), and high-sensitivity C-reactive protein (hsCRP) collectively contribute to the prediction of fatty kidney disease (indicated by solid green lines). Selected metabolic factors—including elevated SBP, uric acid, lipid disturbances, and systemic inflammation—statistically accounted for part of the association between hepatic and renal fat. Dashed yellow arrows denote significant mediation pathways (*p* < 0.05), and standardized coefficients are shown adjacent to the corresponding paths. Created with BioGDP.com.

## 1 Introduction

Ectopic fat deposition, a hallmark of metabolic dysregulation, progressively involves organs beyond adipose tissue, including the liver and kidneys (1, 2). Renal ectopic fat may accumulate in distinct anatomical compartments, including the renal parenchyma, renal sinus, and perirenal region, which can be quantified using the renal fat fraction (RFF), renal sinus fat fraction (RSFF), and perirenal fat fraction (PRFF), respectively (3). Fatty kidney disease (FKD) is increasingly recognized as a distinct clinical entity linked to hypertension (4), decreased glomerular filtration rate (eGFR) (5), and the progression of chronic kidney disease (6). Concurrently, metabolic dysfunction-associated fatty liver disease (MAFLD) has emerged as the most common chronic liver condition worldwide, sharing a common etiological background of insulin resistance, sterile inflammation and metabolic syndrome with FKD (7–9).

Notably, epidemiological and imaging studies frequently report the co-existence of hepatic and renal steatosis, particularly in populations with obesity or type 2 diabetes mellitus (T2DM) (10). Pronounced lipid accumulation has been observed in the kidneys of both high-fat/high-glucose-fed mice and diabetic mouse models (11). This lipid deposition is closely associated with oxidative stress, circulating inflammatory cytokines, and apoptosis (12) that are also known to contribute to hepatocellular injury and hepatic inflammation in the context of MAFLD (13).

As a central metabolic organ, the liver is susceptible to ectopic fat accumulation, which frequently co-exists with renal steatosis in individuals with obesity and T2DM (14). However, it remains unclear whether this coexistence primarily reflects parallel consequences of shared systemic metabolic disturbances or a more direct inter-organ relationship. Previous studies have generally examined hepatic and renal steatosis separately or treated one phenotype as a covariate when evaluating the other. A systematic assessment of their cross-sectional association is therefore warranted. We hypothesized that MRI-derived hepatic fat would be independently associated with renal fat accumulation in adults with T2DM, and that certain metabolic factors might statistically account for part of this association.

Accordingly, this study aimed to quantify the cross-sectional association between MRI-derived hepatic and renal fat fractions, develop and internally validate a model for classifying an exploratory MRI-defined renal steatosis phenotype, and explore metabolic factors that could statistically account for part of the observed relationship.

## 2 Materials and methods

### 2.1 Study population selection

**Figure 1:**
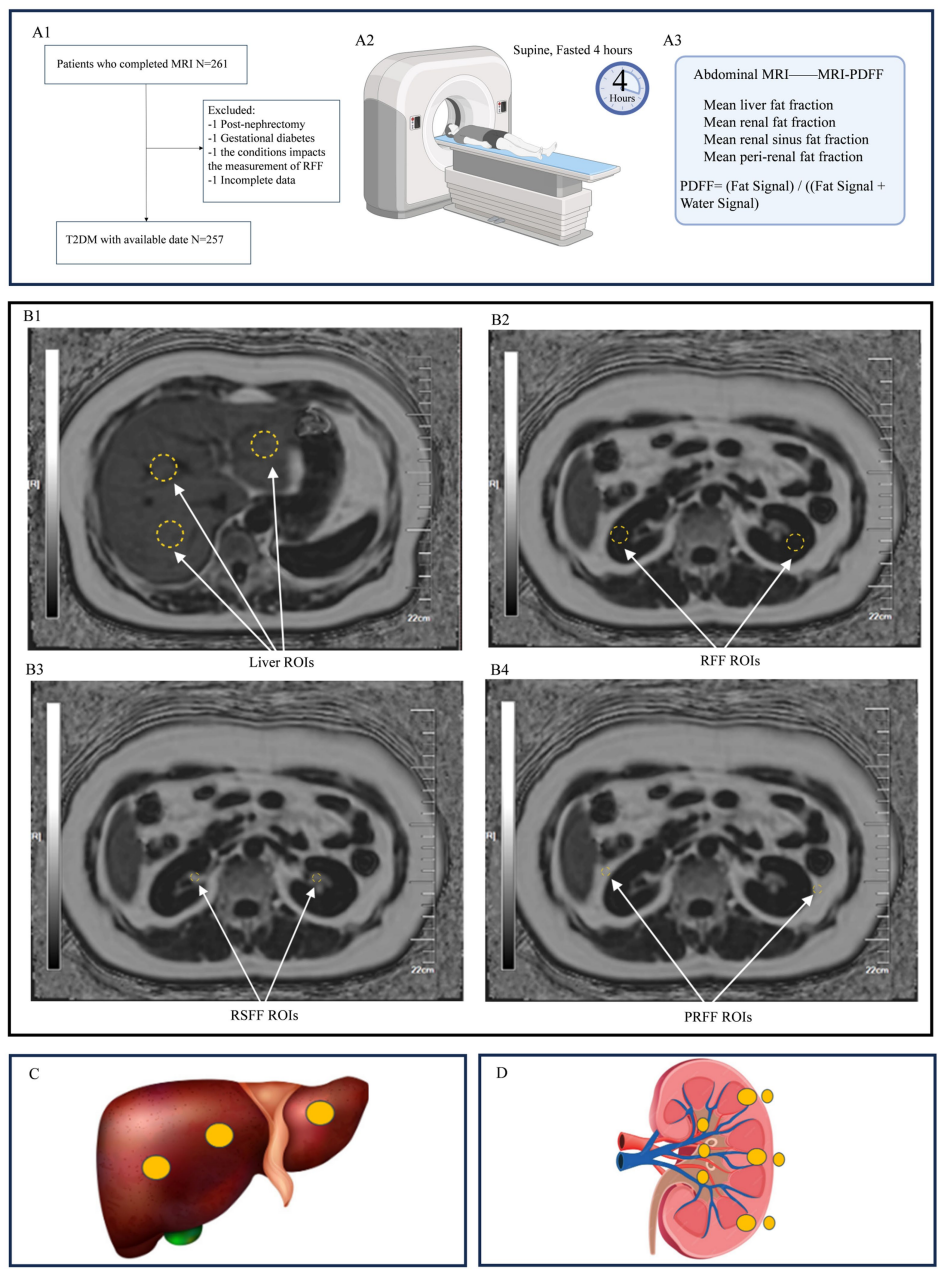
**(A1)** Subject enrollment flow chart. **(A2)** Participants underwent MRI scans in the supine position after a four-hour fast. **(A3)** Patients underwent abdominal MRI, from which average hepatic fat fraction (HFF), renal fat fraction (RFF), renal sinus fat fraction (RSFF), and perirenal fat fraction (PRFF) were recorded. **(B)** A 55-year-old female with a history of type 2 diabetes mellitus. Measurement of **(B1)** HFF and **(B2)** RFF / **(B3)** RSFF / **(B4)** PRFF. **(C,D)**: representative liver **(C)** and kidney **(D)** images with regions of interest (ROIs) for fat quantification. Figure **A2**, **C** and **D** were created in BioRender. z, Z. (2026) https://BioRender.com/kdlrzou.

This cross-sectional study involved 257 participants who were admitted to the Fourth Affiliated Hospital of Soochow University in Suzhou, China, from January 2023 to May 2025. Participants were eligible if they were older than 18 years, diagnosed with T2DM based on the 1999 World Health Organization criteria, and underwent hepatic and renal MRI. The study excluded individuals based on several criteria: those with alcohol addiction (defined as consumption exceeding 140 g/week for males and 70 g/week for females), viral hepatitis, drug-induced liver disease, hepatolenticular degeneration, autoimmune liver disease, other forms of diabetes, acute complications of diabetes, or those suffering from acute infections, active tumors, mental illness, autoimmune disorders, acute kidney injury, or any other confirmed histories of kidney diseases (Figure 1A1).

The study protocols were reviewed and approved by the Medical Ethics Committee of the Fourth Affiliated Hospital of Soochow University, and written informed consent was obtained from all participants.

### 2.2 Metabolic index acquisition

Participants were instructed to fast for 8 to 12 h before sample collection. Venous blood samples were collected from the antecubital vein. Serum samples were obtained by centrifugation at 2,500 rpm for 10 min. Biochemical indexes, including aspartate transaminase (AST), alanine transaminase (ALT), fasting plasma glucose (FPG), fasting C-peptide (FCP), fasting insulin (FINS), serum creatinine (Scr), total cholesterol (TC), triglycerides (TG), high-density lipoprotein cholesterol (HDL-C), low-density lipoprotein cholesterol (LDL-c), and uric acid (UA), were measured using an Mindray automated biochemical analyzer (Shenzhen Mindray Bio-Medical Electronics Co., LTD, Shenzhen, China) at the Clinical Testing Center of the hospital. The urine albumin-to-creatinine ratio (UACR) was measured in first-morning urine specimens. Additionally, glycosylated hemoglobin (HbA1c) was evaluated using a glycosylated hemoglobin analyzer (Mindray H520, China). The chronic kidney disease epidemiology collaboration (CKD-EPI) equation was used to eGFR. Insulin resistance condition was assessed by homeostasis model assessment of insulin resistance (HOMA-IR). HOMA-IR = [FPG (mmol/L) * FINS (mIU/L)]/22.5.

MRI-defined fatty liver was operationally defined as HFF ≥ 5%. Because the present study did not systematically adjudicate all nomenclature-specific criteria for MAFLD or other fatty liver disease frameworks, the term “MRI-defined fatty liver” is used throughout the manuscript. Fatty kidney was defined as RFF ≥ 4%, following the threshold established by Raphael et al. (15). The ≥4% cutoff was adopted as an exploratory working definition for MRI-defined renal steatosis, based on a prior imaging study, solely to facilitate participant stratification and model development. It is not intended to serve as a diagnostic threshold. Sensitivity analyses using thresholds of ≥3% and ≥5% were performed to evaluate the stability of the primary findings. CKD was identified by an eGFR of less than 60 mL/min/1.73 m^2^, calculated using the CKD-EPI formula, and/or an ACR of 30 mg/g or higher (16).

### 2.3 Dixon MRI examination

After a 4-h fast, participants underwent MRI scanning using the multi-echo liver interpolated volume excitation (mLIVE) or IDEAL-IQ method, with imaging volumes centered on the liver and kidneys (Figures 1A2,A3). HFF and RFF maps were automatically reconstructed using the WinStation plug-in algorithm. For hepatic fat quantification, a section located approximately 1 cm above the porta hepatis was selected, and regions of interest (ROIs) were placed in three hepatic segments while avoiding major vessels, bile ducts, and focal lesions (Figures 1B1,C). The mean of the three measurements was recorded as HFF. For renal parenchymal fat quantification, three sections centered on the renal hilum were selected for each kidney, and ROIs were manually delineated on the RFF maps while excluding renal sinus and perirenal adipose tissue (Figures 1B3,B4). RSFF and PRFF were measured separately using three ROIs placed within the corresponding anatomical compartments (Figure 1B2). Measurements from the left and right kidneys were averaged to obtain bilateral RFF, RSFF, and PRFF values (Figure 1D). To assess intra-rater reliability, the same reader re-measured all fat fraction parameters in the entire cohort (*n* = 257) one month after the initial assessment.

### 2.4 Statistical methods

Statistical analyses were performed using SPSS version 27.0 and R Studio 4.5.2, and statistical significance was set at *p* < 0.05. Continuous variables were analyzed for normality using Kolmogorov–Smirnov test and were presented as mean ± standard deviation or median (interquartile ranges). Categorical variables were presented as frequencies and percentages. The relationships between clinical variables and fatty kidney were evaluated using independent samples t-tests, Mann–Whitney U tests, and chi-square tests to compare group means. Participants were classified into four groups according to quartiles of RFF. For evaluating differences in continuous variables across groups, ANOVA, the Kruskal-Wallis test and chi-square analysis were applied. To explore associations between RFF and other clinical measures, the Spearman correlation coefficient was employed. A multiple linear regression analysis was performed to identify independent determinants affecting RFF. Restricted cubic splines (RCS) combined with multivariable linear regression were employed to investigate the nonlinear associations of HFF, FCP, and hsCRP with RFF. Model fitting was implemented using the rms package, and visualization was performed with the ggrcs package.

#### 2.4.1 Model development and internal validation

The final model was developed using LASSO regression with 10-fold cross-validation for variable selection, followed by multivariable logistic regression. Internal validation was performed using 1,000 bootstrap resamples, with the complete modeling procedure (including variable selection and coefficient estimation) repeated in each resample. Optimism was estimated as the mean difference between the performance in each bootstrap sample and that when applying the bootstrap-derived model to the original dataset; the optimism-corrected AUC was then obtained by subtracting this optimism estimate from the apparent AUC. Model discrimination was assessed using the AUC, sensitivity, specificity, positive and negative predictive values, and accuracy; calibration was evaluated using the Brier score. The DeLong test was used to compare the AUCs of two models.

#### 2.4.2 Robustness test

##### 2.4.2.1 Sensitivity analysis under different RFF thresholds

To assess the robustness of the prediction model at different RFF thresholds (≥3% and ≥5%) for fatty kidney, we conducted a sensitivity analysis using R and calculated its AUC.

##### 2.4.2.2 Sensitivity and interaction analyses for FCP

To assess whether renal function confounded the FCP–renal steatosis association, we performed sensitivity analyses by further adjusting the primary model for eGFR or CKD status, and by excluding participants with eGFR < 60 mL/min/1.73 m^2^. We also examined the association in a subgroup of participants not receiving insulin therapy and formally tested for effect modification by including an interaction term between FCP and insulin-treatment status in the multivariable model.

##### 2.4.2.3 XGBoost model verification and SHAP explanation

We interrogated the final extreme gradient boosting (XGBoost) model with SHAP using the shapviz package in R, computing values on the log-odds (margin) scale for the evaluation set (17). Global importance was summarized by mean absolute SHAP and displayed as a full ranked bar plot and as a combined importance and bee swarm summary to show direction and dispersion. Marginal effects and potential interactions for the leading predictors were examined with SHAP dependence plots. For case-level attribution, a waterfall plot decomposed an individual prediction into the model baseline plus signed feature contributions summing to the subject’s log-odds (18).

#### 2.4.3 Exploratory cross-sectional indirect-effect analysis

Exploratory indirect-effect analyses were conducted to quantify the extent to which selected metabolic variables statistically accounted for the cross-sectional association between HFF and RFF. The temporal sequence of HFF → candidate metabolic factor → RFF was specified *a priori* for statistical decomposition, but this temporal ordering could not be verified empirically because all variables were measured simultaneously. Therefore, the estimated indirect effects should not be interpreted as evidence of causal mediation. Bias-corrected 95% confidence intervals were computed using 5,000 bootstrap resamples, and the proportion statistically accounted for was defined as the indirect effect divided by the total effect (19–22).

## 3 Results

### 3.1 Clinical and metabolic parameters of the subjects

Clinical and metabolic parameters of the subjects are shown in Table 1. 257 patients were enrolled, aged 18 to 80 years. Males accounted for 71.6% of the total patients, and the median age and duration of diabetes were 52.00 (38.00,61.50) years and 36.00 (3.00,120.00) months. The average levels of RFF, RSFF, PRFF and HFF were 4.72 (3.45, 5.55) %, 76.90 (70.78, 82.10) %, 85.33 (81.37, 87.92) %, and 8.07 (6.00, 12.37) %, respectively. Intra-rater reliability was excellent for all MRI-derived fat measurements. The ICC for RFF was 0.980 (95% CI, 0.970–0.980; *p* < 0.001). Excellent reproducibility was also observed for HFF (ICC = 0.999, 95% CI, 0.999–0.999), RSFF (ICC = 0.984, 95% CI, 0.979–0.987), and PRFF (ICC = 0.973, 95% CI, 0.965–0.979), with all *p* values <0.001. Of the participants, 108 (42.2%) were untreated with glucose-lowering medications, 13 (5.06%) received metformin monotherapy, 71 (27.63%) were treated with metformin combined with other oral hypoglycemic agents (OHAs), and 44 (17.12%) received insulin therapy with or without OHAs (Supplementary Table S1). In this study, multivariable analysis adjusted only for other medications revealed that lipid-lowering therapy was significantly associated with a lower RFF (Supplementary Table S2). Additionally, 45(17.51%) patients were taking hepatoprotective medications, 81 (31.52%) were on antihypertensive therapy, and 139 (54.09%) were receiving lipid-lowering treatment. Among the participants, 193 (75.10%) had hypertension, 103 (40.08%) exhibited vascular plaques, 77 (29.96%) were obese, and 75 (29.20%) were diagnosed with CKD.

**Table 1:** Baseline demographic and metabolic characteristics of participants, stratified by fatty kidney status.

Variables	All subjects (*n* = 257)	Fatty kidney (*n* = 173)	Non-fatty kidney (*n* = 84)	*p* value
General characteristics				
Male (%)	184 (71.60%)	127 (73.41%)	57 (67.86%)	0.354
Age (year)	52.00 (38.00,61.50)	53.00 (38.50,62.00)	51.00 (37.00,61.00)	0.463
Duration (month)	36.00 (3.00, 120.00)	48.00 (3.00, 120.00)	30.00 (2.00, 87.00)	0.133
BMI (kg/m^2^)	26.12 (23.73,28.81)	26.36 (23.95,29.54)	25.45 ± 4.02	**0.004**
WHR	0.95 (0.91,0.98)	0.95 (0.92,0.98)	0.94 (0.91,0.97)	0.213
SBP (mmHg)	141.00 (131.50,156.00)	146.00 (134.00,159.00)	136.29 ± 20.23	**<0.001**
DBP (mmHg)	88.00 (80.00,95.00)	90.00 (82.00,95.00)	85.82 ± 11.67	**0.038**
Blood glucose metabolism index				
HbA_1c_ (mmol/mol)	91.3 (72.7, 108.8)	91.3 (72.7, 108.8)	90.2 ± 24.6	0.880
FPG (mmol/L)	8.11(6.43,9.87)	8.11 (6.31,10.17)	8.24 ± 2.07	0.312
FINS (mIU/L)	6.66 (1.93, 13.40)	8.60 (1.71, 15.30)	4.88 (2.04, 8.14)	**<0.006**
FCP (ng/mL)	0.94 (0.50, 2.08)	1.14 (0.62, 2.43)	0.66 (0.42, 1.15)	**<0.001**
HOMA-IR	2.20 (0.61, 5.02)	2.74 (0.61, 5.60)	1.65 (0.62, 2.64)	**0.001**
Indicators of renal function and renal injury				
UA (μmol/L)	338.70 (280.60,409.05)	342.60 (298.00,425.80)	319.87 ± 80.92	**0.001**
Scr (μmol/L)	63.00 (51.90, 75.10)	61.10 (50.70, 71.90)	66.92 ± 17.04	0.081
eGFR (mL/min/1.73 m^2^)	106.00 (94.00,117.30)	106.00 (94.50,120.50)	103.24 ± 19.43	0.671
UACR (mg/g)	12.50 (6.54, 32.99)	14.29 (7.29, 36.69)	10.58 (4.62, 19.17)	**0.003**
Liver function index				
ALT (U/L)	20.90 (14.20, 34.50)	21.20 (15.00, 35.10)	18.00 (13.17, 33.23)	0.139
AST (U/L)	20.60 (15.50, 32.00)	20.60 (16.00, 33.10)	20.65 (15.02, 30.75)	0.443
Lipid metabolism index				
TC (mmol/L)	4.86 (4.18,5.51)	4.92 (4.21,5.50)	4.75 ± 1.11	0.161
TG (mmol/L)	1.79 (1.29, 2.76)	1.91 (1.46, 2.79)	1.56 (1.18, 2.52)	**0.008**
LDL-C (mmol/L)	2.97 ± 0.91	3.01 ± 0.91	2.91 ± 0.91	0.398
HDL-C (mmol/L)	0.94 (0.80,1.12)	0.90 (0.79,1.04)	1.03 (0.85,1.34)	**<0.001**
Inflammatory index				
hsCRP (mg/L)	2.32 (1.18, 4.80)	2.74 (1.37, 5.68)	1.59 (0.73, 3.06)	**<0.001**
Visceral fat index				
RFF (%)	4.72 (3.45, 5.55)	——		
RSFF (%)	76.90 (70.78, 82.10)	79.13 (72.30, 83.93)	72.81 (65.19,78.10)	**<0.001**
PRFF (%)	85.33 (81.37, 87.92)	85.92 ± 4.68	81.77 (75.37, 86.37)	**<0.001**
HFF (%)	8.07 (6.00, 12.37)	8.83 (6.70, 13.47)	5.88 (3.28, 10.34)	**<0.001**

For continuous variables following a normal distribution, values are expressed as mean and standard deviation (SD). Non-normally distributed variables are represented as median along with interquartile range. Categorical variables are described using counts and corresponding percentages. Pearson χ^2^ test or Fisher’s exact probability method were used for comparison between disordered groups, and Mann Whitney U test was used for comparison between ordered groups. Bold values indicate statistically significant results.

### 3.2 Correlation and dose–response analyses of RFF with metabolic factors

**Figure 2:**
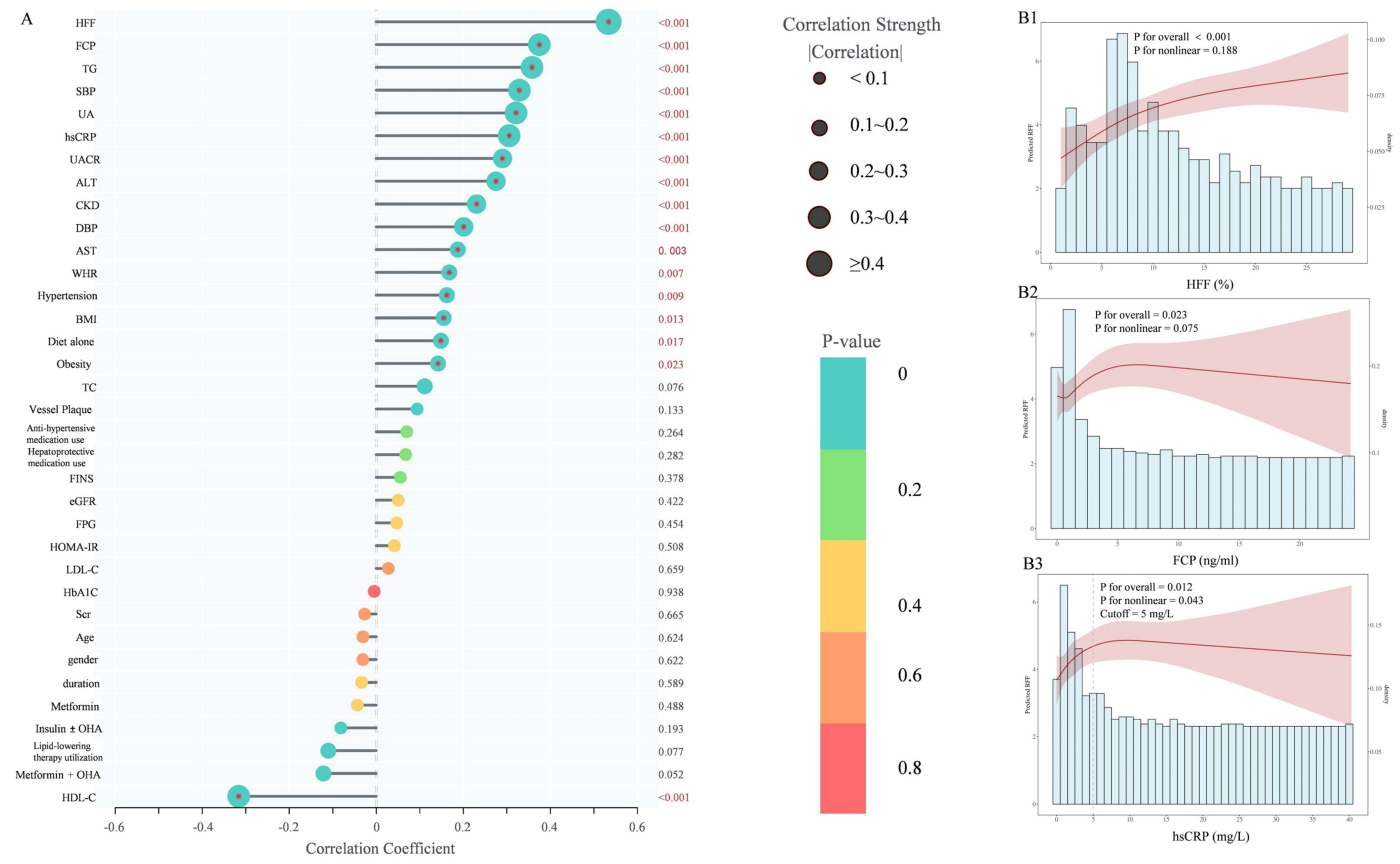
**(A)** Spearman correlation between renal fat fraction (RFF) and metabolic parameters. **(B)** Restricted cubic spline (RCS) analyses of the dose–response relationships of **(B1)** hepatic fat fraction (HFF), **(B2)** fasting C-peptide (FCP), and **(B3)** high-sensitivity C-reactive protein (hsCRP) with RFF. The association for hsCRP was nonlinear (P for nonlinearity <0.05), with an inflection point at approximately 5 mg/L. Models were adjusted for age, sex, BMI, and other covariates. Model for HFF adjusted for gender, age, duration + BMI, WHR, SBP, DBP, diabetes pharmacotherapy, hepatoprotective medication use, antihypertensive medication use and lipid-lowering therapy utilization, HbA1C, FPG, FCP, TG, HDL, UA, hsCRP; Model for FCP adjusted for gender, age, duration + BMI, WHR, SBP, DBP, diabetes pharmacotherapy, hepatoprotective medication use, antihypertensive medication use and lipid-lowering therapy utilization, HbA1C, FPG, HFF, TG, HDL, UA, hsCRP; Model for hsCRP adjusted for gender, age, duration + BMI, WHR, SBP, DBP, diabetes pharmacotherapy, hepatoprotective medication use, antihypertensive medication use and lipid-lowering therapy utilization, HbA1C, FPG, FCP, TG, HDL, UA, HFF.

Then, we further analyzed the correlation between the indicators and RFF in diabetic patients. In Figure 2A, we found that RFF was positively correlated with SBP, FCP, UA, TG, hsCRP, and HFF (r = 0.329, 0.375, 0.321, 0.358, 0.305, 0.534, respectively, *p* < 0.001). While only HDL-C was negatively correlated with RFF (r = −0.316, p < 0.001). With the increase of RFF quartile, SBP, DBP, FCP, UA, UACR, AST, ALT, TG, hsCRP, RSFF, PRFF and HFF showed an increasing trend, while HDL-C showed a decreasing trend (Supplementary Table S3). Restricted cubic spline (RCS) regression was performed to examine the shape of the associations between key metabolic factors and RFF (Figures 2B1–B3). HFF exhibited a linear relationship with RFF (P for nonlinearity = 0.188). Similarly, no evidence of nonlinearity was observed for FCP (P for nonlinearity >0.05). In contrast, hsCRP demonstrated a significant nonlinear association with RFF (P for nonlinearity = 0.043). Piecewise linear regression identified an optimal inflection point at 5 mg/L. In participants with hsCRP ≤5 mg/L, hsCRP was positively associated with RFF (*β* = 0.208, 95% CI: 0.076–0.341, *p* = 0.002). However, no significant association was observed when hsCRP exceeded 5 mg/L (β = −0.008, 95% CI: −0.067–0.050, *p* = 0.786).

### 3.3 Multiple linear regression analysis of the influencing factors of RFF in patients with T2DM

Multivariable linear regression analysis identified HFF, FCP, and hsCRP as independent correlates of RFF. In the model with adjustment for age, gender, and diabetes duration, HFF was positively associated with RFF (*β* = 0.172, 95% CI: 0.138–0.205, *p* < 0.001). After sequential adjustment for blood pressure and medication use (Model 2), metabolic parameters (Model 3), and liver enzymes, insulin resistance, and complications (Model 4), the association remained significant, although the effect size was attenuated (Model 4: β = 0.110, 95% CI: 0.066–0.154, *p* < 0.001). The positive associations of FCP and hsCRP with RFF were robust across all models and remained significant after full adjustment (FCP: β = 0.099, 95% CI: 0.025–0.173, *p* < 0.01; hsCRP: β = 0.041, 95% CI: 0.008–0.074, *p* < 0.05). Variance inflation factors for all variables were below 10, indicating no significant multicollinearity (see Table 2).

**Table 2:** Multiple linear regression analysis of the influencing factors of RFF in T2DM patients.

Variables	Model 1 β (95% CI)	Model 2 β (95% CI)	Model 3 β (95% CI)	Model 4 β (95% CI)
HFF (%)	0.172 (0.138, 0.205)***	0.146 (0.111, 0.181) ***	0.097 (0.057, 0.137) ***	0.110 (0.066, 0.154) ***
FCP (ng/mL)	0.187 (0.115, 0.259) ***	0.144 (0.073, 0.215) ***	0.092 (0.021, 0.163)*	0.099 (0.025, 0.173)**
hsCRP (mg/L)	0.072 (0.033, 0.112) ***	0.054 (0.016, 0.091)**	0.041 (0.007, 0.074)*	0.041 (0.008, 0.074)*

Dependent variable: RFF. Model 1 adjusted for gender, age, duration; Model 2 adjusted for Model 1 + BMI, WHR, SBP, DBP, Diabetes pharmacotherapy, Hepatoprotective medication use, Antihypertensive medication use and Lipid-lowering therapy utilization; Model 3 for HFF adjusted for Model 2 + HbA1C, FPG, FCP, TG, HDL, UA, hsCRP; Model 3 for FCP adjusted for Model 2 + HbA1C, FPG, HFF, TG, HDL, UA, hsCRP; Model 3 for hsCRP adjusted for Model 2 + HbA1C, FPG, FCP, TG, HDL, UA, HFF. Model 4 adjusted for Model 3 + ALT, AST, HOMAIR, Vvessel plaque, Obesity. All variables included in the linear regression analysis had a VIF less than 10.

### 3.4 Baseline data and metabolic characteristics grouped according to the presence or absence of fatty kidney

Although fatty kidney disease has been studied in general populations, we focused our analysis on patients with T2DM to explore its implications in a high-risk group. This decision was motivated by the increasing recognition that metabolic disorders (23, 24), particularly diabetes (25), may alter the pathophysiology of kidney fat deposition, thus distinguishing it from the general population. By examining diabetic patients, we sought to explore whether the presence of fatty kidney in this group is associated with more pronounced metabolic disturbances or kidney dysfunction, which may not be evident in the broader population. We found significant differences between the two groups in BMI, SBP, DBP, FINS, FCP, HOMA-IR, UA, UACR, TG, HDL-C, hsCRP, RSFF, PRFF and HFF (Table 1).

### 3.5 Development and internal validation of the classification model for MRI-defined renal steatosis

**Figure 3:**
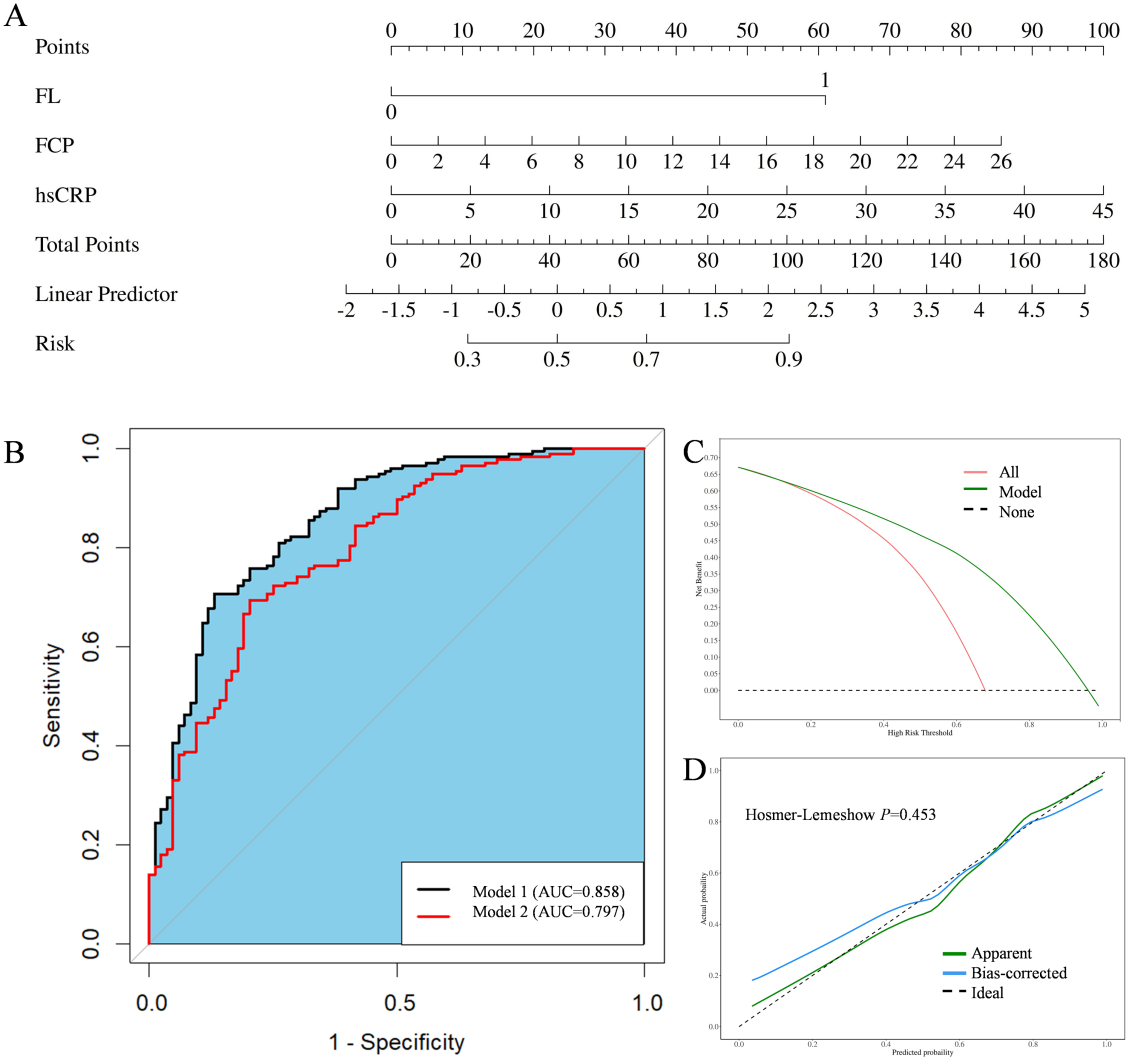
Development and internal evaluation of the logistic classification model for MRI-defined renal steatosis. **(A)** The nomogram includes FL (fatty liver), FCP, and hsCRP. **(B)** Model 1 demonstrated an AUC of 0.858, indicating a good discriminatory ability for predicting RFF. Model 2 demonstrated an AUC of 0.797. **(C)** Decision curve analysis shows that under a specific threshold, this logistic regression model has a good application value for clinical decision-making. **(D)** The calibration curve indicates that the model has good calibration performance in predicting the risk of fatty kidney. Hosmer-Lemeshow test *p* > 0.05.

35 variables measured at hospital admission (Table 1; Supplementary Table S1) were included in the LASSO regression. LASSO regression retained 25 variables with nonzero coefficients (Supplementary Figure S1). three variables remained independently associated with MRI-defined renal steatosis in the multivariable logistic model: fatty liver, FCP, and hsCRP (Supplementary Figure S2). These variables were used to construct a predictive model (Figure 3). Specifically, FCP (*β* = 0.16, OR (95%CI) = 1.17 (1.01–1.36), *p* < 0.05), hsCRP (β = 0.10, OR (95%CI) = 1.10 (1.01–1.20), p < 0.05), and fatty liver (β = 2.37, OR (95%CI) = 10.70 (3.65–33.88), *p* < 0.001) were significantly associated with an higher odds of meeting the RFF-based renal steatosis criterion. These predictors were used to develop a nomogram for estimating the probability of fatty kidney in patients with T2DM (Figure 3A). To further evaluate the performance of this model (Model 1), several validation analyses were performed. ROC curve analysis (Figure 3B) demonstrated that the model 1 showed an apparent AUC of 0.858 (95% CI, 0.808–0.907). After 1,000 bootstrap resamples, the optimism-corrected AUC was 0.833, corresponding to an optimism estimate of 0.025. To assess the contribution of fatty liver to the prediction of fatty kidney, an alternative logistic regression model (Model 2) was constructed, excluding fatty liver and including only FCP and hsCRP. The diagnostic performance of Model 2 was then compared with that of Model 1 (which included fatty liver) using ROC curve analysis. The AUC for Model 2 was 0.797(95% CI: 0.739–0.855), which was significantly lower than that of Model 1 (*p* = 0.004), indicating a significantly lower discrimination after omission of MRI-defined fatty liver. Model 1 achieved an overall accuracy of 0.759 (95% CI: 0.702–0.810), with a sensitivity of 0.869 (95% CI: 0.797–0.941) and a specificity of 0.705 (95% CI: 0.637–0.773). The positive predictive value (PPV) was 0.589 (95% CI: 0.502–0.675), while the negative predictive value (NPV) was 0.917 (95% CI: 0.870–0.964). The optimal probability threshold determined by the Youden index was 0.754. Additionally, DCA (Figure 3D) shows that under a specific threshold, model 1 has a good application value for clinical decision-making. Finally, the Calibration Curve (Figure 3D) showed agreement between the predicted and observed probabilities of meeting the MRI-defined renal steatosis criterion within the development cohort. The logistic equation we obtain is:


$$\begin{array}{c} \text{Logit}\;[P(\mathrm{FKD}=1)]=-2.68+2.37\times \text{fatty liver}+0.16\times \mathrm{FCP}\\ +0.10\times \text{hsCRP} \end{array}$$

### 3.6 Robustness test

#### 3.6.1 Sensitivity analysis under different RFF thresholds

We assessed model performance across three RFF thresholds (≥3%, ≥4%, and ≥5%) as sensitivity analyses. At the ≥4% primary threshold, the model achieved an AUC of 0.858 (95% CI: 0.808–0.907). At ≥3%, 211 participants (82.1%) were classified as positive; the model yielded an AUC of 0.827 (95% CI: 0.714–0.867), with sensitivity of 0.744, specificity of 0.761, and a Brier score of 0.114. At the primary threshold of RFF ≥ 4%, 173 participants (67.3%) were classified as positive. At ≥5%, 92 participants (35.8%) were positive; the AUC was 0.784 (95% CI: 0.648–0.826), with sensitivity of 0.837, specificity of 0.661, and a Brier score of 0.190. Positive and negative predictive values varied substantially across thresholds due to differences in prevalence, ranging from 0.579 to 0.935 for PPV and 0.393 to 0.879 for NPV, while discrimination (AUC) remained more stable (Supplementary Figure S3).

#### 3.6.2 Sensitivity and interaction analyses for FCP

Additional adjustment for renal function did not materially alter the FCP effect estimate. Adjustment for eGFR yielded an OR of 1.14 (95% CI: 1.00–1.31; *p* = 0.0585); adjustment for CKD status gave an OR of 1.14 (95% CI: 0.99–1.30; *p* = 0.0699); and excluding participants with eGFR < 60 mL/min/1.73 m^2^ produced a similar estimate (OR = 1.14, 95% CI: 0.99–1.31; *p* = 0.0607). In an exploratory subgroup analysis of participants not receiving insulin therapy, the OR was 1.26 (95% CI: 1.04–1.53; *p* = 0.0162). However, the formal interaction test between FCP and insulin treatment was not statistically significant (coefficient = −0.250; *p* = 0.085), providing insufficient evidence of effect modification. Overall, the point estimates remained consistent across renal-function adjustments, although the confidence intervals overlapped the null, indicating limited statistical precision.

#### 3.6.3 XGBoost model verification and SHAP explanation

**Figure 4:**
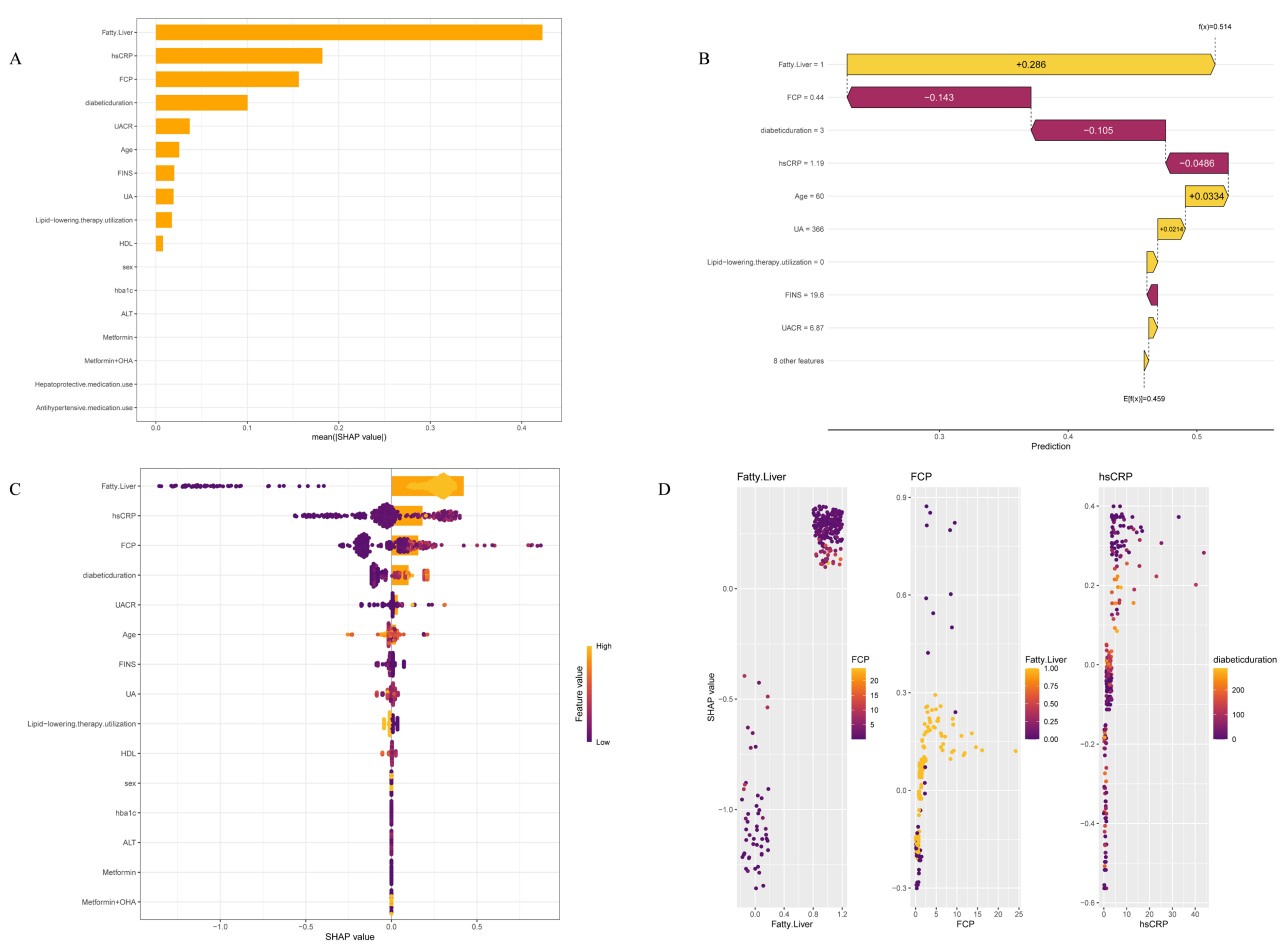
Complementary exploratory XGBoost and SHAP analyses. **(A)** Global SHAP feature importance for the XGBoost model. **(B)** Individual-level SHAP waterfall plot. **(C)** SHAP global importance and summary beeswarm for the XGBoost model. **(D)** SHAP dependence plots for the top predictors (fatty liver, FCP, and hsCRP) in the XGBoost model. The coloring variable represents the strongest interactions automatically identified by the model.

In the XGBoost model, fatty liver, FCP, and hsCRP were the top contributors by mean absolute SHAP (Figure 4A). A representative waterfall plot (Figure 4B) illustrated their contributions to the model output for an individual participant. The importance and summary bee swarm (Figure 4C) and dependence plots (Figure 4D) indicated a discrete positive shift for fatty liver and increasing nonlinear contributions for FCP and hsCRP. These findings align with the predictors retained in the logistic model.

### 3.7 Comparison between isolated hepatic steatosis and coexisting hepatic and renal steatosis

Considering the previously established close association between fatty liver and fatty kidney, we further compared baseline characteristics between patients with simple fatty liver (H1) and those with coexisting fatty liver and fatty kidney (H2) to further characterize the metabolic profiles associated with coexisting hepatic and renal steatosis. The age, diabetes duration, SBP, FINS, FCP, HOMA-IR and hSCRP of H2 group were significantly higher than those of H1 group (*p* < 0.05). The prevalence of hypertension was significantly higher in the H2 group compared to the H1 group (Supplementary Table S4).

### 3.8 Exploratory cross-sectional indirect-effect analysis

**Figure 5:**
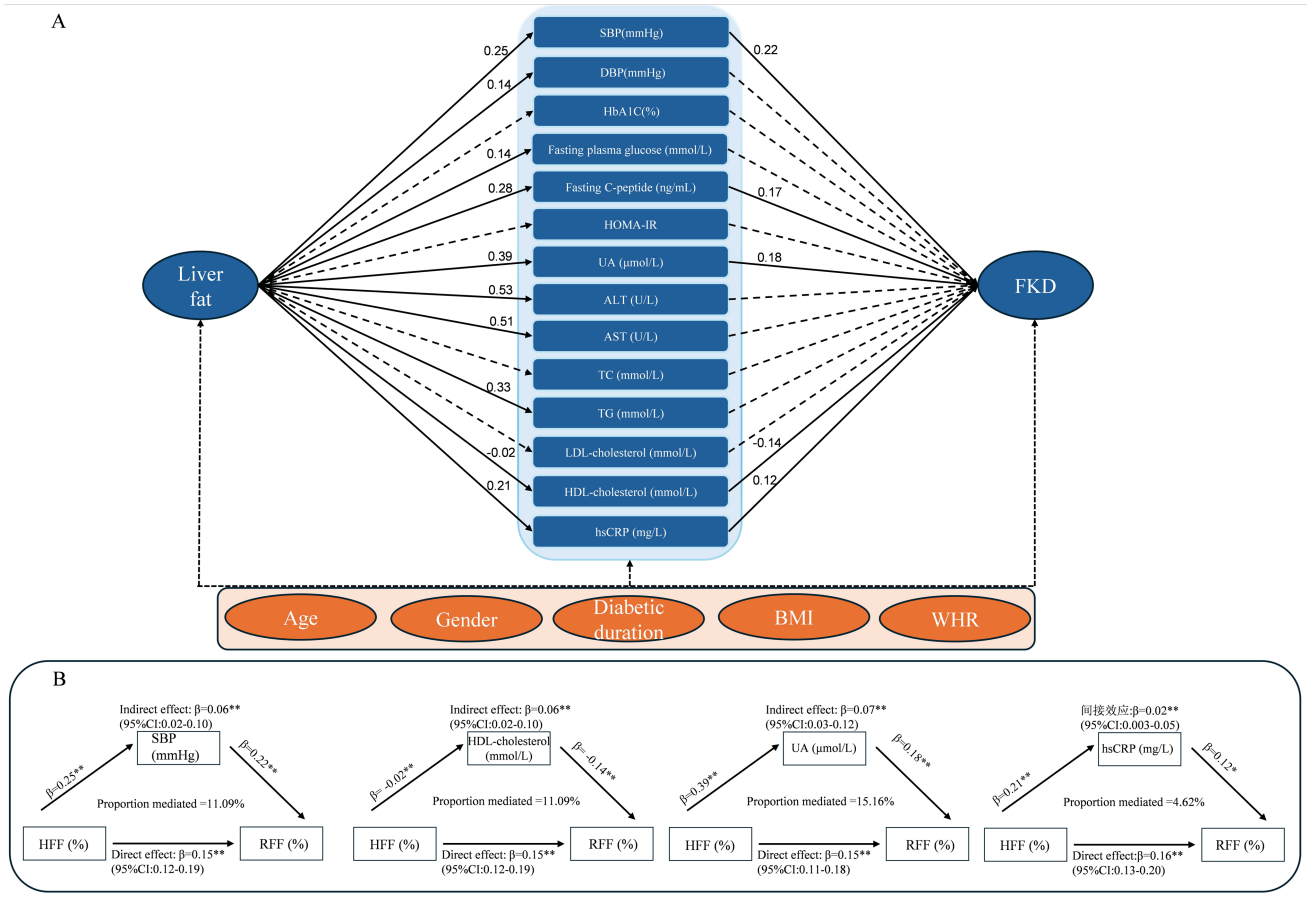
**(A)** Effects of liver fat on metabolic traits and of metabolic traits on fatty kidney. **(B)** Mediation model diagram of SBP, HDL-cholesterol, UA, and hsCRP in the association between HFF and RFF in patients with type 2 diabetes. The arrows denote prespecified statistical paths and should not be interpreted as evidence of temporal ordering or causal mediation.

Exploratory indirect-effect analyses were performed for 14 candidate metabolic and inflammatory variables (Supplementary Table S5). Statistically significant indirect-effect estimates were observed for SBP, UA, HDL-C, and hsCRP. The estimated indirect effects were 0.06 (95% CI, 0.02–0.10) for SBP, 0.07 (95% CI, 0.03–0.12) for UA, 0.06 (95% CI, 0.02–0.10) for HDL-C, and 0.02 (95% CI, 0.003–0.05) for hsCRP. These factors statistically accounted for 11.09, 15.16, 11.09, and 4.62% of the total HFF–RFF association, respectively (Supplementary Table S6; Figure 5). These estimates represent cross-sectional statistical decompositions and do not establish causal mediation.

## 4 Discussion

In this cross-sectional study of adults with T2DM, we identified a strong association between MRI-derived hepatic and renal fat accumulation. Two principal findings emerged. First, a parsimonious classification model incorporating MRI-defined fatty liver, FCP, and hsCRP showed good discrimination for an exploratory RFF-defined renal steatosis phenotype, with the AUC decreasing only modestly from 0.858 to an optimism-corrected value of 0.833 after bootstrap validation. Nevertheless, because the model was developed in a single-center cohort, external validation is required before clinical application. Second, exploratory indirect-effect analyses suggested that SBP, UA, HDL-C, and hsCRP statistically accounted for modest proportions of the observed HFF–RFF association. Collectively, these findings support the coexistence of coordinated hepatic and renal ectopic fat phenotypes in T2DM, while not establishing temporal direction, causal mediation, or a direct organ-to-organ pathway.

The parsimonious model incorporating MRI-defined fatty liver, FCP, and hsCRP showed good apparent discrimination for the exploratory RFF-defined renal steatosis phenotype and retained acceptable discrimination after bootstrap internal validation. Decision-curve analysis suggested potential net benefit within the evaluated range of threshold probabilities, although this finding should not be interpreted as evidence of established clinical utility. At the primary RFF threshold, the model showed a relatively high negative predictive value but a modest positive predictive value, suggesting that it may be more useful for identifying individuals unlikely to meet the MRI-defined renal steatosis criterion than for confirming its presence. Sensitivity analyses using RFF thresholds of ≥3% and ≥5% yielded AUCs of 0.827 and 0.784, respectively, indicating acceptable but threshold-dependent discrimination. In a complementary exploratory XGBoost analysis, SHAP-based feature importance also ranked MRI-defined fatty liver as the leading contributor, followed by FCP and hsCRP, supporting concordance between the nonlinear and logistic modeling approaches. Nevertheless, external validation is required before the model can be considered for clinical risk stratification or used to guide decisions regarding further imaging assessment (40).

MRI-defined fatty liver was associated with approximately tenfold higher odds of meeting the exploratory renal steatosis criterion. This strong cross-sectional association does not, however, establish that hepatic fat accumulation directly causes renal fat deposition, as the two phenotypes may share common systemic determinants—including insulin resistance, adipose tissue dysfunction, circulating free fatty acids, inflammation, medication exposure, and genetic susceptibility. Nevertheless, our finding is consistent with previous reports. In a cross-sectional imaging study, individuals with fatty liver were more likely to have renal steatosis (15), and an 18-month randomized trial (DIRECT, NCT01530724) showed that reductions in renal fat paralleled hepatic metabolic improvements rather than direct renal functional changes (26). Moreover, the lower AUC of the model excluding MRI-defined fatty liver suggests that hepatic fat provided incremental discriminatory information beyond routine metabolic variables. Although the mechanisms linking hepatic and renal steatosis remain incompletely understood, potential pathways—including gut microbiota dysbiosis, lipid transport mediators, and altered adipokine signaling—have been proposed. Using exploratory mediation analysis, we identified several metabolic factors that may statistically account for a modest proportion of the observed association. Nonetheless, given the cross-sectional design, longitudinal studies with repeated organ-specific fat measurements are required to establish temporal direction and clarify the biological underpinnings of this hepato-renal network.

The indirect-effect estimates for UA, SBP, HDL-C, and hsCRP were statistically significant but modest, each accounting for approximately 5–15% of the observed HFF–RFF association. These variables may represent interconnected metabolic domains—purine metabolism, hemodynamic regulation, lipid transport, and systemic inflammation—rather than principal mediating pathways. For instance, elevated UA may promote renal lipogenesis via SIRT1 suppression (27, 28), consistent with evidence that MAFLD is associated with higher UA levels (29). SBP elevation may activate renin–angiotensin–aldosterone signaling (7, 30), while HDL-C dysfunction may impair reverse cholesterol transport from renal cells (31, 32). However, these mechanistic interpretations remain speculative given the cross-sectional design. Notably, the majority of the HFF–RFF association remained unexplained, suggesting potential contributions from unmeasured factors such as systemic free-fatty-acid flux, adipokines, hepatokines, oxidative stress, altered renal fatty-acid oxidation, and gut microbiota-related signaling. Future studies incorporating direct measures of these pathways are needed to clarify the biological underpinnings of the hepato-renal crosstalk.

HsCRP statistically accounted for the smallest proportion of the association (4.62%), suggesting that systemic inflammation may explain only a limited component of the observed HFF–RFF relationship (33, 34). Although liver enzymes were correlated with RFF in unadjusted analyses, their indirect-effect estimates were not statistically significant. This observation suggests that hepatic fat content and hepatocellular injury may reflect distinct metabolic dimensions—the former representing lipid accumulation and the latter indicating hepatocyte inflammation or necrosis—and that only the fat-related dimension was statistically associated with the HFF–RFF relationship in our mediation framework. However, this should not be interpreted as evidence that HFF is a superior causal risk indicator; rather, it highlights the need for longitudinal studies with paired measurements of hepatic fat, liver enzymes, and renal outcomes to disentangle these pathways.

Fasting C-peptide (FCP), a marker of endogenous insulin secretion, was identified as a variable associated with MRI-defined renal steatosis. Elevated FCP may reflect compensatory hyperinsulinemia, which could promote ectopic lipid accumulation through insulin-stimulated lipogenesis. However, FCP should not be interpreted as a direct measure of insulin resistance, and its circulating levels are influenced by renal clearance. In sensitivity analyses, the FCP effect estimates remained similar in magnitude after adjustment for eGFR, CKD status, or exclusion of participants with reduced eGFR (OR ≈ 1.14 across models), although the corresponding confidence intervals overlapped the null (*p* = 0.058–0.070), indicating limited statistical precision. An exploratory subgroup analysis suggested a stronger association among participants not receiving insulin therapy (OR = 1.26), but the formal interaction test was not significant (*p* = 0.085), precluding a definitive conclusion of effect modification. Collectively, these findings indicate that FCP is a potentially informative metabolic correlate of renal steatosis, but its role should be considered exploratory and requires confirmation in longitudinal studies with direct measures of insulin sensitivity and secretion (7, 35–38).

Previous studies have demonstrated that excessive lipid deposition, a hallmark feature of diabetic nephropathy in both human and animal models, correlates with renal injury through mechanisms involving lipid accumulation, lipotoxicity, and lipid metabolic dysregulation (12). In our cohort, however, CKD prevalence did not differ significantly according to renal steatosis status. This discrepancy may partly reflect the relatively preserved renal function (mean eGFR within the normal range) in our participants, which contrasts with the broader spectrum of kidney impairment in other study populations (39). Despite the absence of an association with overt CKD, RFF showed a positive correlation with UACR—a marker of early glomerular injury—consistent with the notion that lipotoxicity may promote subclinical renal damage (33, 34, 37). Whether renal steatosis represents an early pathogenic event that precedes functional decline, or merely a marker of systemic metabolic burden, remains to be determined. Longitudinal studies with repeated measurements of renal fat and function are therefore needed to clarify the temporal relationship between renal steatosis and progressive kidney injury.

This study has several limitations. First, its cross-sectional design precludes determination of temporal direction or causal relationships among hepatic fat, metabolic factors, and renal fat. Accordingly, the indirect-effect analyses represent statistical decompositions under an assumed ordering rather than evidence of causal mediation. Second, the RFF threshold of 4% was used as an exploratory working definition and has not been established as a universal diagnostic criterion. Variations in study populations, MRI acquisition protocols, reconstruction algorithms, and ROI placement may affect renal fat estimates and threshold performance. Third, the classification model was developed in a relatively small, single-center cohort without independent external validation. Although bootstrap validation suggested only modest optimism, its transportability to other populations remains uncertain. Residual confounding is also possible because dietary intake, physical activity, smoking, alcohol consumption below the exclusion threshold, socioeconomic factors, and detailed use of individual glucose-lowering medications were not fully captured. In addition, the cohort consisted exclusively of Chinese adults with T2DM, limiting generalizability to other ethnic groups and individuals without diabetes. Circulating C-peptide is influenced by renal clearance, and although renal-function-adjusted analyses yielded similar point estimates, statistical precision was limited. Finally, the indirect-effect analyses were exploratory, inter-rater reproducibility was not assessed, and the model requires further evaluation before application to hepatic steatosis identified using other imaging modalities or clinical criteria.

In this MRI-based cross-sectional study of adults with T2DM, hepatic steatosis was strongly associated with renal fat accumulation. A three-variable model incorporating MRI-defined fatty liver, FCP, and hsCRP showed good internally validated discrimination for an exploratory renal steatosis phenotype, although independent external validation remains necessary. SBP, UA, HDL-C, and hsCRP statistically accounted for modest proportions of the observed HFF–RFF association in cross-sectional indirect-effect analyses. These findings provide a practical framework for identifying individuals at risk for renal steatosis and generate testable hypotheses for future longitudinal and mechanistic studies.

## Data Availability

The original contributions presented in the study are included in the article/Supplementary material, further inquiries can be directed to the corresponding author.
