# Supplementary material for: Association of hepatic steatosis with MRI-defined renal steatosis in type 2 diabetes: a cross-sectional imaging and clinical modeling study
Source: Front Nutr. 2026 Jul 31;13:1881517. doi: 10.3389/fnut.2026.1881517 (PMC13474305; doi:10.3389/fnut.2026.1881517)
Supplement: Supplementary file 1 [file Supplementary_file_1.DOCX]

**Supplemental Table S1. Clinical Comorbidities and Medication Profile Stratified by Fatty Kidney Status.**

| **Variables** | **All subjects (n=257)** | **Fatty Kidney(n=173)** | **Non-Fatty Kidney(n=84)** | **P value** |
| --- | --- | --- | --- | --- |
| **Diabetes pharmacotherapy** | |  |  |  |
| **None(diet alone)** | 108(42.20%) | 80(46.24%) | 28(33.33%) | **0.049** |
| **Metformin** | 13(5.06%) | 7(4.05%) | 6(7.14%) | 0.448 |
| **Metformin +OHA** | 71 (27.63%) | 41 (23.70%) | 30 (35.71%) | **0.043** |
| **Insulin ±OHA** | 44(17.12%) | 25 (14.45%) | 19 (22.62%) | 0.103 |
| **Hepatoprotective medication use** | 45 (17.51%) | 35 (20.23%) | 10 (11.90%) | 0.099 |
| **Antihypertensive medication use** | 81 (31.52%) | 60 (34.68%) | 21 (25.00%) | 0.117 |
| **Lipid-lowering therapy utilization** | 139 (54.09%) | 89 (51.45%) | 50 (59.52%) | 0.223 |
| **Comorbidities** |  |  |  |  |
| **Hypertension, n(%)** | 193 (75.10%) | 138 (79.77%) | 55 (65.48%) | **0.013** |
| **Vessel Plaque, n(%)** | 103 (40.08%) | 75 (43.35%) | 28 (33.33%) | 0.124 |
| **Obesity, n(%)** | 77 (29.96%) | 57 (32.95%) | 20 (23.81%) | 0.134 |
| **CKD, n(%)** | 75 (29.20%) | 56 (32.40%) | 19 (22.60%) | 0.095 |

Note: OHA: oral hypoglycemic agents; CKD: Chronic kidney disease.

**Supplemental Table S2. Associations Between Antidiabetic Medications and RFF in Univariate and Multivariable Regression Analyses.**

| **Variables** | **Univariate** | | | | **Multivariable** | | | |
| --- | --- | --- | --- | --- | --- | --- | --- | --- |
|  | **β** | **S.E** | **t** | **P** | **β** | **S.E** | **t** | **P** |
| **None(diet alone)** | 0.516 | 0.226 | 2.280 | **0.023** | 0.239 | 0.303 | 0.788 | 0.431 |
| **Metformin** | 0.318 | 0.514 | 0.618 | 0.537 | 0.362 | 0.556 | 0.651 | 0.516 |
| **Metformin+OHA** | -0.529 | 0.250 | -2.116 | **0.035** | -0.372 | 0.308 | -1.206 | 0.229 |
| **Insulin±OHA** | 0.431 | 0.298 | 1.444 | 0.150 | 0.232 | 0.329 | -1.206 | 0.229 |

Dependent variable: RFF. All variables included in the linear regression analysis had a VIF less than 5.

**Supplemental Table S3. Baseline data and metabolic characteristics grouped by quartiles of RFF.**

| **Variables** | **Q1(n=65)** | **Q2(n=64)** | **Q3(n=64)** | **Q4(n=64)** | **P for trend** |
| --- | --- | --- | --- | --- | --- |
| **General characteristics** | | |  |  |  |
| **SBP(mmHg)** | 135.05±18.50 | 141.64±20.31 | 144.95±18.23* | 153.38±18.83**## | **<0.001** |
| **DBP(mmHg)** | 86.37±11.49 | 86.28±12.28 | 87.19±12.05 | 93.58±16.88*# | **0.005** |
| **Blood glucose metabolism index** | | | |  |  |
| **FINS (mIU/L)** | 4.55 (1.94, 6.67) | 9.54 (3.92, 13.58)* | 11.95 (5.30, 19.38)** | 4.36 (0.62, 12.45)## | **<0.001** |
| **FCP (ng/mL)** | 0.64(0.40, 1.18) | 0.85 (0.44, 1.46) | 0.95 (0.59, 1.71) | 2.03 (1.07, 4.34)**##^^ | **<0.001** |
| **HOMA-IR** | 1.45 (0.55, 2.32) | 3.10 (1.32, 5.42)** | 3.60 (1.83, 7.52)** | 1.54 (0.26, 5.37)#^^ | **<0.001** |
| **Indicators of renal function and renal injury** | | | |  |  |
| **UA (μmol/L)** | 320.64±74.02 | 331.59±92.99 | 343.67±82.91 | 398.85±105.38**##^^ | **<0.001** |
| **UACR (mg/g)** | 10.26(4.36, 17.16) | 11.14 (5.67, 20.11) | 13.04 (7.48, 34.65) | 18.85 (8.34, 64.32)**# | **0.002** |
| **Liver function index** | | |  |  |  |
| **ALT (U/L)** | 17.70 (12.90, 33.30) | 18.55 (14.57, 29.05) | 18.90 (13.17, 28.52) | 30.60 (20.43, 49.70)**##^^ | **<0.001** |
| **AST (U/L)** | 20.40 (14.60, 30.00) | 20.00 (15.02, 30.20) | 18.80 (15.80, 28.13) | 28.70 (18.95, 42.85)*#^ | **0.012** |
| **Lipid metabolism index** | | |  |  |  |
| **TG (mmol/L)** | 1.60 (1.13, 2.67) | 1.56 (1.28, 2.16) | 1.74 (1.28, 2.20) | 2.65 (2.07, 3.64) **##^^ | **<0.001** |
| **HDL-C (mmol/L)** | 1.15±0.38 | 0.99±0.28* | 0.98±0.26* | 0.89±0.19** | **<0.001** |
| **Inflammatory index** | | |  |  |  |
| **hsCRP(mg/L)** | 1.57 (0.88, 3.35) | 1.91 (0.93, 3.04) | 3.29 (1.58, 5.77)**## | 3.24 (1.69, 6.11)**## | **<0.001** |
| **Visceral fat index** | | |  |  |  |
| **RSFF(%)** | 70.74±9.39 | 74.95±8.27 | 76.16±8.49** | 81.44±7.93**##^^ | **<0.001** |
| **PRFF(%)** | 80.01±7.74 | 84.04±5.66** | 84.13±4.47**## | 88.22±4.21**## | **<0.001** |
| **HFF(%)** | 5.90 (2.97, 10.57) | 6.35 (5.59, 7.31) | 8.62 (7.75, 10.14)**## | 14.05 (9.91, 17.74)**##^^ | **<0.001** |

Note: All subjects were divided into four groups(Q1/Q2/Q3/Q4) according to RFF quartiles. Differences among groups were tested using the one-way analysis of variance (ANOVA) test or Kruskal-Wallis’one-way ANOVA test for continuous variables, and χ2 test for categorical variables. *P<0.05, and **P<0.01 vs Q1 group; #P<0.05, and ##P<0.01 vs Q2 group; ^P<0.05,^^P<0.01 vs Q3 group (Statistical significance based on Tukey’s HSD post-hoc test and the Bonferroni correction).

**Supplemental Table S4. Basic characteristic of 207 fatty liver patients.**

| **Variables** | **H1(n=47)** | **H2(n=160)** | **P value** |
| --- | --- | --- | --- |
| **General characteristics** |  |  |  |
| **Age (year)** | 46.02±14.68 | 50.99±14.74 | **0.027** |
| **Duration (month)** | 12.00 (2.00, 60.00) | 36.00 (3.00, 120.00) | **0.002** |
| **SBP(mmHg)** | 139.28±19.52 | 147.09±19.00 | **0.009** |
| **Blood glucose metabolism index** | |  |  |
| **FINS (mIU/L)** | 5.33 (2.57, 6.67) | 9.54 (2.77, 16.07) | **0.040** |
| **FCP (ng/mL)** | 0.67 (0.48, 1.28) | 1.12 (0.62, 2.15) | **0.021** |
| **HOMA-IR** | 1.78 (0.97, 2.53) | 3.10 (1.07, 5.75) | **0.008** |
| **Indicators of renal function and renal injury** | | |  |
| **UA (μmol/L)** | 337.30±84.80 | 365.00±96.69 | **0.001** |
| **UACR(mg/g)** | 10.66 (5.58, 16.91) | 14.25 (7.24, 34.78) | **0.050** |
| **Inflammatory index** |  |  |  |
| **hsCRP(mg/L)** | 1.65 (0.85, 3.27) | 2.89 (1.38, 5.79) | **＜0.001** |
| **Complicating other diseases** | |  |  |
| **hypertension ,n(%)** | 29 (63.04%) | 129 (80.62%) | **0.013** |

Note: We defined H1 as the fatty liver group and H2 as the combination of fatty liver and fatty kidney group. Pearson's χ² test or Fisher's exact test was used for comparing categorical variables between groups, while the Kruskal-Wallis H test and one way Anova was applied for comparing continuous variables between groups.

**Supplemental Table S5. Effects of liver fat on metabolic traits and of metabolic traits on fatty kidney.**

| **Trait** | **Standard coefficient** | **SE** | **p value** |
| --- | --- | --- | --- |
| **Fatty liver and metabolic traits** | | | |
| **Effect of liver fat on SBP** | 0.25 | 0.21 | **<0.001** |
| **Effect of liver fat on DBP** | 0.14 | 0.15 | **0.03** |
| **Effect of liver fat on HbA1C** | -0.01 | 0.07 | 0.85 |
| **Effect of liver fat on FPG** | 0.14 | 0.03 | **0.03** |
| **Effect of liver fat on FCP** | 0.28 | 0.03 | **<0.001** |
| **Effect of liver fat on HOMAIR** | -0.01 | 0.06 | 0.84 |
| **Effect of liver fat on UA** | 0.39 | 0.96 | **<0.001** |
| **Effect of liver fat on ALT** | 0.53 | 0.18 | **<0.001** |
| **Effect of liver fat on AST** | 0.51 | 0.17 | **<0.001** |
| **Effect of liver fat on TC** | 0.07 | 0.01 | 0.28 |
| **Effect of liver fat on TG** | 0.33 | 0.02 | **<0.001** |
| **Effect of liver fat on LDL** | -0.08 | 0.01 | 0.24 |
| **Effect of liver fat on HDL** | -0.02 | 0.003 | **<0.001** |
| **Effect of liver fat on hsCRP** | 0.21 | 0.06 | **0.002** |
| **Metabolic traits and fatty kidney** | | | |
| **Effect of SBP on fatty kidney** | 0.22 | 0.01 | **<0.001** |
| **Effect of DBP on fatty kidney** | 0.10 | 0.01 | 0.07 |
| **Effect of HbA1C on fatty kidney** | 0.07 | 0.02 | 0.19 |
| **Effect of FPG on fatty kidney** | 0.14 | 0.04 | 0.44 |
| **Effect of FCP on fatty kidney** | 0.17 | 0.03 | **0.002** |
| **Effect of HOMAIR on fatty kidney** | 0.01 | 0.02 | 0.13 |
| **Effect of UA on fatty kidney** | 0.18 | 0.001 | **0.002** |
| **Effect of ALT on fatty kidney** | -0.02 | 0.01 | 0.78 |
| **Effect of AST on fatty kidney** | -0.03 | 0.01 | 0.69 |
| **Effect of TC on fatty kidney** | 0.09 | 0.09 | 0.09 |
| **Effect of TG on fatty kidney** | 0.11 | 0.05 | 0.06 |
| **Effect of LDL on fatty kidney** | 0.06 | 0.11 | 0.27 |
| **Effect of HDL on fatty kidney** | -0.14 | 0.35 | **0.02** |
| **Effect of hsCRP on fatty kidney** | 0.12 | 0.02 | **0.03** |

Single-mediator model with single-level data was used to investigate mediation. All mediation analyses were adjusted for age, gender, WHR, diabetic duration and BMI.

**Supplemental Table S6. Indirect and direct effect estimates with bootstrap Cis.**

| **Effect** | **Estimated coefficient(95%CI)** | **Proportion mediated (%)** | **Standardized indirect effect(95%CI)** |
| --- | --- | --- | --- |
| **Direct effect on fatty kidney** | 0.15(0.12,0.19) | - | - |
| **Indirect effect through SBP** | 0.02(0.01,0.03) | 11.09% | **0.06(0.02,0.10)** |
| **Direct effect on fatty kidney** | 0.16(0.13, 0.20) | - | - |
| **Indirect effect through DBP** | 0.004(-0.001,0.01) | - | 0.01(-0.003,0.03) |
| **Direct effect on fatty kidney** | 0.17(0.13,0.20) | - | - |
| **Indirect effect through HbA1c** | -0.0003(-0.002,0.002) | - | -0.001(-0.0074,0.006) |
| **Direct effect on fatty kidney** | 0.17(0.13,0.20) | - | - |
| **Indirect effect through FPG** | 0.002(-0.003,0.01) | - | 0.01(-0.01,0.03) |
| **Direct effect on fatty kidney** | 0.15(0.12,0.19) | - | - |
| **Indirect effect through FCP** | 0.01(-0.0003,0.04) | - | 0.05(-0.001,0.10) |
| **Direct effect on fatty kidney** | 0.17(0.13,0.20) | - | - |
| **Indirect effect through Homa-IR** | 0.00(-0.002,003) | - | -0.0001(-0.0054,0.009) |
| **Direct effect on fatty kidney** | 0.15(0.11,0.18) | - | - |
| **Indirect effect through UA** | 0.02(0.01,0.04) | 15.16% | **0.07(0.03,0.12)** |
| **Direct effect on fatty kidney** | 0.17(0.13,0.21) | - | - |
| **Indirect effect through ALT** | -0.003(-0.03,0.03) | - | -0.01(-0.11,0.10) |
| **Direct effect on fatty kidney** | 0.17(0.13,0.21) | - | - |
| **Indirect effect through AST** | -0.004(-0.03,0.02) | - | -0.01(-0.10,0.06) |
| **Direct effect on fatty kidney** | 0.17(0.13,0.20) | - | - |
| **Indirect effect through TC** | 0.002(-0.002,0.01) | - | 0.01(-0.01,0.02) |
| **Direct effect on fatty kidney** | 0.16(0.12,0.19) | - | - |
| **Indirect effect through TG** | 0.01(0.0002,0.02) | - | **0.04(0.001,0.08)** |
| **Direct effect on fatty kidney** | 0.17(0.14,0.20) | - | - |
| **Indirect effect through LDL** | -0.001(-0.006,0.001) | - | -0.005(-0.02,0.004) |
| **Direct effect on fatty kidney** | 0.15(0.12,0.19) | - | - |
| **Indirect effect through HDL** | 0.02(0.01,0.03) | 11.09% | **0.06(0.02,0.10)** |
| **Direct effect on fatty kidney** | 0.16(0.13,0.20) | - | - |
| **Indirect effect through hsCRP** | 0.01(0.001,0.02) | 4.62% | **0.02(0.003,0.05)** |

The single-mediator model with single-level data was used. All mediation analyses were adjusted for age, gender, WHR, diabetic duration and BMI. Proportion mediated represents an estimate of the extent to which the direct effect was accounted for by the pathway through the mediating variable. Significance of mediation was tested by computing bias-corrected bootstrap confidence intervals with 5000 repetitions.

**Supplemental Figure S1.** LASSO Regression for Feature Selection. After LASSO regression selection, 25 variables remained significant predictors of fatty kidney, including demographics (gender, age, diabetic duration, DBP), metabolic parameters (BMI, WHR, HbA1c, FCP, TG, HDL), clinical factors (fatty liver, HFF, AST, eGFR, UA), inflammatory marker (hsCRP), comorbidities(vessel plaque, CKD， hyper tension) and medication use (metformin monotherapy, metformin combined with OHAs, insulin therapy with or without OHAs, hepatoprotective, lipid-lowering therapies, and antihypertensive medication use).

**Supplemental Figure S2.** Forest Plot of Multivariable Logistic Regression Analysis for Predictors of Fatty Kidney Disease. Given their established roles as potential confounders, dura (diabetes duration) and metformin combined with other OHAs were treated as adjustment variables in the analysis and were not considered for inclusion as independent predictors in the final model.

**Supplemental Figure S3.** Diagnostic performance of predictive models at different kidney fat thresholds: AUCs of 0.827 at 3%, 0.858 at 4%, and 0.784 at 5% cutoff points.
